# Decreased Camptothecin Sensitivity of the Stem-Cell-Like Fraction of Caco2 Cells Correlates with an Altered Phosphorylation Pattern of Topoisomerase I

**DOI:** 10.1371/journal.pone.0099628

**Published:** 2014-06-24

**Authors:** Amit Roy, Cinzia Tesauro, Rikke Frøhlich, Marianne S. Hede, Maria J. Nielsen, Eigil Kjeldsen, Bjarne Bonven, Magnus Stougaard, Irina Gromova, Birgitta R. Knudsen

**Affiliations:** 1 Department of Molecular Biology and Genetics, Aarhus University, Aarhus, Denmark; 2 Zymonostics ApS, Aarhus, Denmark; 3 Hemodiagnostic Laboratory, Cancercytogenetic Section, Aarhus University Hospital, Aarhus, Denmark; 4 Department of Pathology, Aarhus University Hospital, Aarhus, Denmark; 5 Genome Integrity Unit, Proteomics in Cancer, Danish Cancer Research Center, Danish Cancer Society, Copenhagen, Denmark; Florida International University, United States of America

## Abstract

The CD44+ and CD44− subpopulations of the colorectal cancer cell line Caco2 were analyzed separately for their sensitivities to the antitumor drug camptothecin. CD44+ cells were less sensitive to camptothecin than CD44− cells. The relative resistance of CD44+ cells was correlated with (i) reduced activity of the nuclear enzyme topoisomerase I and (ii) insensitivity of this enzyme to camptothecin when analyzed in extracts. In contrast, topoisomerase I activity was higher in extracts from CD44− cells and the enzyme was camptothecin sensitive. Topoisomerase I from the two subpopulations were differentially phosphorylated in a manner that appeared to determine the drug sensitivity and activity of the enzyme. This finding was further supported by the fact that phosphorylation of topoisomerase I in CD44+ cell extract by protein kinase CK2 converted the enzyme to a camptothecin sensitive, more active form mimicking topoisomerase I in extracts from CD44− cells. Conversely, dephosphorylation of topoisomerase I in extracts from CD44− cells rendered the enzyme less active and camptothecin resistant. These findings add to our understanding of chemotherapy resistance in the Caco2 CD44+ cancer stem cell model.

## Introduction

Colorectal cancer is among the most common human malignancies and one of the leading causes of cancer deaths [1]. Metastatic colorectal cancer remains incurable with the available systemic therapeutic options. However, during the past decade new combinational therapies including derivatives of the plant alkaloid camptothecin (CPT), such as irinotecan or topotecan have shown very promising results [2], [3]. Despite the very encouraging results only a subset of colorectal cancer patients respond to the CPT-based therapies and problems with primary resistance or relapsed tumors remain a major problem [4], [5], [6], [7].

CPT and its derivatives act by interfering with the activity of the nuclear human enzyme topoisomerase I (TopI), which has been identified as the sole cellular target of these drugs [8], [9], [10].

TopI relaxes topological tension in the genome by cleaving-religating one strand in the DNA double helix. Under normal circumstances the enzyme leaves the DNA intact [11]. However, selective inhibition of the religation reaction of TopI catalysis in the presence of CPT leads to the accumulation of transient TopI-bound nicks in genomic DNA [12], [13], [14]. Upon collision with DNA-tracking processes e.g. transcription and replication these TopI-DNA complexes are converted into permanent DNA damage that ultimately can result in cell death [14], [15], [16]. The cytotoxicity of CPTs correlates directly with the intracellular activity of TopI and common mechanisms behind CPT resistance in cell lines include down-regulation of TopI activity [17], [18], [19], [20], [21], [22], [23] or mutations in the *TOP1* gene leaving the enzyme insensitive towards CPT [4], [24], [25], [26], [27], [28], [29]. The importance of the cell-to-cell heterogeneity (intratumor heterogeneity) characteristic for most cancer cell populations for the occurrence of drug resistance is less well characterized [30], [31].

During recent years the cancer stem cell (CSC) theory has been proposed to explain the origin and progression of a variety of cancers including colorectal cancer and bulk evidence now support this theory [30], [32], [33], [34], [35], [36], [37], [38]. CSCs are defined as a population of cancer cells with tumor initiating capacity. They can undergo indefinite self-renewal and have the capacity to differentiate into the diverse cell populations constituting the bulk of a tumor. Today CSCs are considered to engender tumor genesis, metastasis and therapeutic resistance of most cancers [39], [40], [41]. Several features of CSCs make them refractory to current treatment strategies. In the *in vivo* tumors CSCs are relatively quiescent. Moreover, they have been proposed to exhibit high expression levels of multi-drug transporter proteins [42] and DNA damage response genes [39], [43]. Consequently, CSCs may be selectively enriched and capable of regenerating tumor growth after chemotherapeutic treatments.

CSCs are often identified and isolated on the basis of specific surface markers. In the current study we have taken advantage of the colorectal cancer cell line Caco2. This cell line has previously been demonstrated to sustain a subpopulation of CSCs with tumor initiating capacity characterized by the expression of the CD133 and CD44 surface markers even when grown in serum containing media [44]. The non-CSC subpopulation of Caco2 does not express CD44 (CD44−), allowing for physical sorting of the two cell populations. We observed a CPT resistant phenotype of the CD44+ cell subpopulation, which was not shared by the CD44− cell subpopulation. The CPT resistant phenotype could be ascribed directly to the phosphorylation status of TopI expressed in CD44+ cells leading to reduced enzyme activity and rendering the enzyme insensitive to CPT when analyzed in extract from these cells.

## Materials and Methods

### Reagents and Enzymes

Phi29 DNA polymerase was purchased from MBI Fermentas. CodeLink Activated Slides were purchased from SurModics Inc. (USA), and Vectashield was from Vector Laboratories Inc. (USA). CD44 MicroBeads (human), FcR Blocking Reagent (human), CD44-PE Antibody (human), CD133-PE Antibody (human), Casein Kinase II (CK2) and alkaline phosphatase from calf intestine were purchased from New England BioLabs Ltd. (UK). Pap Pen was purchased from Dako (Glostrup, Denmark).

MTT (3-(4,5-Dimethylthiazol-2-yl)-2,5-Diphenyltetrazolium Bromide) reagent (M5655) was purchased from Sigma-Aldrich ApS (Denmark), dissolved in phenol red MEM at a final concentration of 5 mg/ml, sterilized using 0.22 µm filter and stored at −20°C.

### REEAD substrates, primers and probes

The sequences of the oligonucleotides are as follows:

S(hTopI): 5′-AGAAAAATTT TTAAAAAAAC TGTGAAGATC GCTTATTTTT TTAAAAATTT TTCTAAGTCT TTTAGATCCC TCAATGCTGC TGCTGTACTA CGATCTAAAA GACTTAGA-amine-3′ [45]

Oligonucleotide for generation of control circles (control ligase substrate): 5′-p-AGAAAAATTTTTAAAAAAACTGTGAAGATCGCTTATTTTTTTAAAAATTTTTCTAAGTCTTTTAGATC-CCGAGATGTACCGCTATCGTCAT-GATCTAAAAGACTT-3′

RCA primer (Anti-TopI primer): 5′-amine-CCAACCAACCAACCAAATAAGCGATCTTCACAGT-3′

ID16: TAMRA-CCTCAATGCTGCTGCTGTACTAC

IDA1: 6′FAM-CCGAGATGTACCGCTATCGT

All oligonucleotides were purchased from DNA Technology A/S (Denmark).

The detection probes ID16 for detection of rolling circle amplification products (RCP) generated from circularized S(hTopI) and IDA1 for detection of RCPs generated from control circles were fluorescently labeled by 5′-coupling of the fluorophores TAMRA and 6′FAM, respectively. For preparation of the control circles, the control ligase substrate was circularized by T4 DNA ligase, and the resulting ligated circles were purified from a 8% polyacrylamide gel. The concentration of the obtained circles was determined by spectrophotometric measurement [45].

### Cells Culture

Caco2 cell culture obtained from ATCC was grown in MEM supplemented with 20% fetal bovine serum (FBS), 1% non-essential amino acids (NEAA), 100 units/ml penicillin and 100 µg/ml streptomycin (Invitrogen A/S, Denmark). The cell cultures were maintained in a humidified incubator (5% CO_2_/95% air atmosphere at 37°C). Cells were plated into tissue culture dishes (Nunc, Roskilde, Denmark) at an initial density of 6×10^4^ cells/cm^2^ in all the experiments except the MTT assay.

Cells were harvested by trypsin treatment (0.05% Trypsin-EDTA solution, Sigma-Aldrich ApS, Denmark) followed by two consecutive washes with 1×PBS and stored at −80°C until further analysis.

### FACS analysis and cell sorting

Caco2 cell subpopulations were counted or separated into two fractions by FACS using a Gallios Flow Cytometer (Beckman Coulter Danmark ApS) after staining with CD44-PE or CD133-PE antibodies. For cell sorting the cells were kept cold and sorted using a FACSAria III (BD Biosciences, San Jose, CA) with a 561 nm laser to excite both PE and PI. Light emitted from PE was collected using a 582/15 band pass filter while light emitted from PI was collected using a 610/20 band pass filter. Doublets and dead cells were excluded and CD44 PE negative and positive cells were sorted. The CD44 positive gate was defined as the most positive 10% of the population. FlowJo software (v.9.6.2, TreeStar Inc., Ashland, OR) was used for data analysis. For each experiment, the background fluorescence was measured using both unlabeled cell mixture and cell mixture labeled with the appropriate concentration of PE-conjugated antibodies. After each FACS sorting the enriched fractions were analyzed for CD44+ cells. In all cases FACS separation resulted in at least 94% pure CD44+ and 99% pure CD44− cell populations, respectively.

### Cell survival assay of CD44− and CD44+ cells

CPT sensitivity of the two cell populations was determined by MTT colorimetric assay. Sorted cells (CD44− or CD44+) were seeded in 96 well tissue culture plates (Nunc Roskilde, Denmark) at a concentration of 9×10^3^ cells/cm^2^ in 100 µl of growth media. After 24 hours the growth media was removed and replaced with 100 µl fresh growth media containing dimethyl sulfoxide (DMSO) or CPT at different concentrations, as stated in the text. After 60 hours of treatment 10 µl of MTT (5 mg/ml) was added to each well and incubation was continued in the incubator (5% CO_2_/95% air atmosphere at 37°C) for 4 hours in the dark. After incubation, the 96 well tissue culture plate containing the samples was centrifuged at 1000 g for 5 minutes and medium was removed. The precipitated formazan was dissolved with DMSO and the absorbance of each well analyzed at 590 nm using a FLUOstar OPTIMA (BMG Labtech, Ortenberg, Germany) microplate reader. The results were graphically represented by subtracting the absorbance of DMSO alone (as blank) and normalizing the 590 nm absorbance of CPT treated cells to the absorbance of DMSO treated cells (as negative control) for both CD44− and CD44+ cells.

### Preparation of whole cell extract and nuclear extract

Cells were harvested by treatment with 0.05% Trypsin solution (Sigma-Aldrich, Denmark A/S). For preparation of whole cell extracts that was used in all REEAD experiments, 2×10^4^ cells were lysed by mixing the pelleted cells with 10 µl lysis Buffer A (10 mM Tris, pH 7.5, 5 mM EDTA, 0.1 mM phenylmethyl sulfonyl fluoride (PMSF) followed by 1 minute incubation on ice. This procedure resulted in lysis of both cell- and nuclear membranes.

Nuclear extracts were used for TopI purification, SDS-PAGE and Western blot analyses. Nuclear extracts were prepared by incubation of cells with lysis buffer B (0.1% NP-40, 10 mM Tris, pH 7.9, 10 mM MgCl_2_, 15 mM NaCl, 1 mM DTT and, 0.1 mM PMSF) on ice for 10 minutes. Subsequently, the nuclei were pelleted by centrifugation at 400 g for 10 minutes. The pelleted nuclei were extracted by addition of 100 µL nuclear extraction buffer (0.5 M NaCl, 20 mM HEPES, pH 7.9, 20% glycerol, 1 mM DTT and, 0.1 mM PMSF) followed by rotation for 1 hour at 4°C [46]. Cell debris was removed by centrifugation at 9000 g for 10 minutes at 4°C.

### Detection of TopI activity by REEAD assay

The REEAD assay was performed essentially as previously described [45]. The 5′-amine-coupled RCA primer was linked to CodeLink Activated Slides according to the manufacturer's description. The TopI reactions were carried out in a 20 µL reaction volume containing a divalent cation depletion buffer (10 mM Tris-HCl, pH 7.5, 5 mM EDTA and 50 mM NaCl). The reaction mixtures were supplemented with 500 nM S(hTopI) DNA substrate. Reactions were initiated by the addition of cell extract or purified TopI in the presence or absence of CPT as indicated in the figure legends. Incubation was continued for 30 minutes at 37°C before heat inactivation of enzymes present in the reaction mixture for 5 minutes at 95°C. Subsequently, hybridization to the surface coupled primer was performed for 60 minutes at room temperature (22 to 25°C). To allow normalization, 5 nM of control circles were added to the heat inactivated reaction mixture prior to this step. Slides were washed for 2 minutes at room temperature in wash buffer 1 (0.1 M Tris-HCl, pH 7.5, 150 mM NaCl, and 0.3% SDS) and subsequently for 2 minutes at room temperature in wash buffer 2 (0.1 M Tris-HCl, pH 7.5, 150 mM NaCl, and 0.05% Tween-20). Finally, the slides were dehydrated in 99.9% ethanol for 1 minute and air-dried. Rolling circle DNA synthesis was performed for 45 minutes at 37°C in 1× Phi29 buffer supplemented with 0.2 µg/µL BSA, 250 µM dNTP, and 1 unit/µL Phi29 DNA polymerase. The reaction was stopped by washing with wash buffers 1 and 2, the slides were dehydrated in 99.9% ethanol for 1 minute and air-dried. The rolling circle products were detected by hybridization to 0.17 µM of each of the detection probes ID16 (red fluorescent labeled) and IDA1 (green fluorescent labeled) in a buffer containing 20% formamide, 2× SSC, and 5% glycerol for 30 min at 37°C. The slides were washed in wash buffers 1 and 2, dehydrated, mounted with Vectashield, and visualized under fluorescence microscope as described previously. For quantitative depiction of the results the number of signals originating from TopI activity (red signals) or spike in control circles (green signals), respectively, were counted on 12 microscopic images and the results calculated as the ratio between the number of red (R) and green (G) signals (R/G) as described in [45].

### Purification of endogenous TopI from CD44− and CD44+ cells

Nuclear extracts were prepared from 5×10^6^ FACS sorted CD44+ or CD44− cells as described above, except that phosphatase inhibitor cocktail 1 and 2 (Sigma-Aldrich, Denmark) were added to all buffers at a final concentration of 10 µL/mL to prevent dephosphorylation of TopI. The extract was loaded onto a 50 µL nickel-NTA-agarose column (Qiagen Nordic, Denmark), pre-equilibrated with buffer containing 10 mM Tris-HCl, pH 7.5, 300 mM NaCl, 5 mM MgCl_2_, 10% Glycerol, and 1 mM PMSF. The column was washed with 1 mL wash buffer containing 10 mM Tris-HCl, pH 7.5, 300 mM NaCl, 10% Glycerol, 20 mM imidazole, pH 7.8 and 1 mM PMSF. Bound protein was eluted from the column with 500 µL elution buffer containing 10 mM Tris-HCl, pH 7.5, 300 mM NaCl, 5 mM MgCl_2_, 10% Glycerol, 150 mM imidazole, pH 7.8 and 1 mM PMSF and collected in 50 µL fractions. A protein purity of approximately 50% was obtained, as gauged by SDS-polyacrylamide gel-electrophoresis (SDS-PAGE) followed by silver staining performed according to manufacturers protocol (Invitrogen A/S, Denmark). Fractions with equal concentrations of TopI were used for REEAD analysis.

### Phosphorylation and dephosphorylation of TopI

Dephosphorylation was performed by incubation of whole cell extract from 10^4^ CD44− or CD44+ cells with 5 units of alkaline phosphatase at 37°C for 30 minutes in the reaction buffer containing 50 mM Tris-HCl (pH 9.3 at 25°C), 1 mM MgCl_2_, 0.1 mM ZnCl_2_ and 1 mM spermidine.

Phosphorylation was performed by incubation of whole cell extract from 10^4^ CD44− or CD44+ cells with 250 units of protein kinase CK2 at 30°C for 10 minutes [44] in the reaction buffer containing 20 mM Tris-HCl (pH 7.5 at 25°C), 50 mM KCl, 10 mM MgCl_2_, and 200 µM ATP.

### Polyacrylamide electrophoresis (PAGE) and Western blotting

The two-dimensional PAGE (2D PAGE) was carried out using an immobilized pH gradient (IPG) as previously described [47]. Briefly, the IPG strips pI 3–10, were actively rehydrated with 200 µl of sample solution containing proteins from approximately 0.5×10^6^ cells. The isoelectrofocusing was carried out for 18,000 Vhr. using the Bio-Rad PROTEAN IEF (Bio-Rad laboratories, Denmark). The second dimension was carried out as previously described [48]. All samples were run twice. The separated proteins were visualized by silver nitrate staining according to the procedure compatible with mass spectrometry (MS). Silver stained gels were dried between cellophane followed by MS analysis.

The separation of proteins by one dimensional SDS-PAGE gel was performed by using NuPAGE system (Invitrogen A/S, Denmark) according to manufacturer's instructions. In brief, cell lysates were mixed with NuPAGE LDS loading buffer and NuPAGE sample reducing agent. Samples were then incubated at 70°C for 10 min and loaded onto NuPAGE Novex 4–12% Bis-Tris gels with 50 µg/lane. The protein separation was performed in NuPAGE MOPS buffer containing NuPAGE antioxidant according to manufacturer's instructions.

Western blotting was performed according to procedure described elsewhere [49]. The resolved proteins were blotted onto Hybond-C nitrocellulose membranes (Amersham Biosciences, USA) and reacted with a TopI specific rabbit antibody (1∶2000 TopI antibody, Epitomics, USA) followed by detection of immune complexes with a horseradish peroxidase-labeled polymer (1∶200) (Envision+ detection kit; DAKO; Denmark). The blots were developed using the Amersham ECL plus Western Blotting Detection Kit (GE Health/Amersham Bioscience, VWR, Denmark) according to manufacturer's instructions. The signals were visualized with a CCD camera (BioSpectrum Imaging System, UVP BioImaging, Upland, CA, USA). The membranes were additionally reversibly stained with Ponceau S solution (Sigma-Aldrich, Denmark) to match location of proteins in membrane with the Western blot signal and to ensure a proper focusing of protein spots.

For 2D gel-electrophoretic analyses dephosphorylation was performed as described: 1×10^6^ CD44+ or CD44− cells were lysed in the presence of 1 mM NaCl/0.4% NP40 and divided into two aliquots. One aliquot of the CD44+ or CD44− cells were subjected to dephosphorylation performed by treatment with the lambda protein phosphatase (λ-PPase) (1600 units) in λ-PPase buffer at 30°C for 45 minutes according to manufacturer's instructions (New England, BioLabs Inc., USA). (Note, that two different phosphatases were used in present study. However, since the role of both phosphatases was merely to remove all phosphorylations such experimental discrepancy does not influence the final results). One aliquot of each cell fraction was mock-treated as a control. The reactions were stopped by freezing the samples at −80°C followed by lyophilization. The samples were then solubilized in CLB1 lysis buffer (R&D Systems, Inc., USA) and subjected to 2D PAGE and 2D Western blotting.

## Results

### Decreased CPT sensitivity of CD44+ cells relative to CD44− cells of Caco2 correlates with decreased activity and decreased CPT susceptibility of TopI in CD44+

The colorectal cancer cell line Caco2 has previously been demonstrated to contain a subpopulation of CSCs with tumor initiating capacity characterized by expression of the CD133 and CD44 surface markers when grown in serum containing media [44].

**Figure 1:**
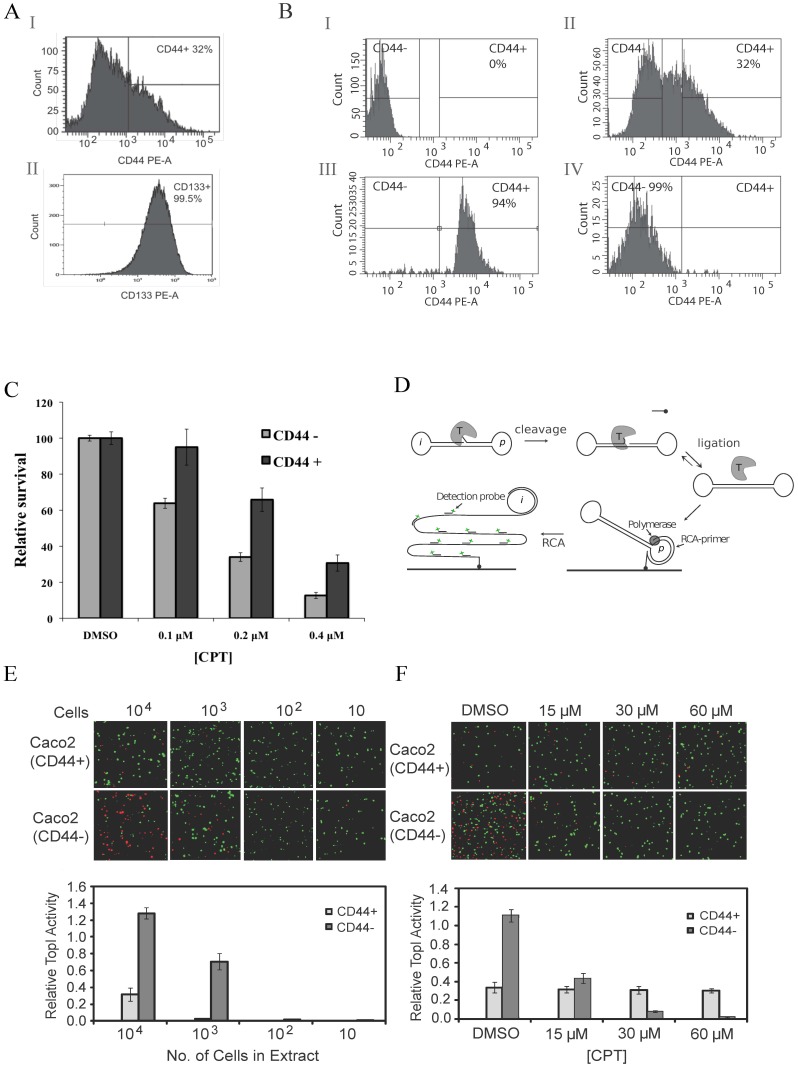
CPT response of CSC or non-CSC cell subpopulations of Caco2. (**A**) FACS analysis of Caco2 cells stained with PE-conjugated CD44 (top panel) or CD133 (lower panel) specific antibodies. (**B**) FACS analysis of Caco2 cells before or after FACS sorting based on CD44. (I) Unstained cells, (II) Caco2 cells stained for CD44 before sorting, (III) Caco2 cells after sorting for CD44+ cells, (IV) Caco2 cells after sorting for CD44− cells. The cells were analyzed after sorting by staining for CD44 (**C**) Survival assay of the FACS sorted CD44+ or CD44− cells. The cells were incubated with DMSO (the solvent of CPT) or 0.1, 0.2, or 0.4, µM of CPT for 60 hours before the percentage of viable cells was measured by the MTT method. (**D**) Graphical depiction of the REEAD assay. S(hTopI) folds into a dumbbell shaped structure, which supports cleavage and ligation activity mediated by TopI. Hereby the substrate is converted to a closed circle, which is hybridized to a surface-attached RCA-primer matching the specific primer annealing sequence (p) on the substrate. Subsequently, phi-polymerase is added to support RCA. The resulting RCA products are visualized by hybridization of fluorescently labeled probes matching the complementary identifier sequence (i) of the circles and the products analyzed using a fluorescence microscope. TopI is illustrated as a gray pacman with a “T”, the phi-polymerase as a dark gray circle. The RCA-primers and detection probes are indicated on the figure. (**E**) Measurement of TopI activity by REEAD in the CD44+ and CD44− FACS sorted cell populations, respectively. Representative microscopic images obtained by analyzing the TopI activity present in whole cell extracts from 10-10^4^ cells are shown in the top panel. Signals obtained from circularized S(hTopI) are shown in red, while signals obtained from control circles are shown in green. The lower panel shows a quantitative depiction of three independent experiments. (**F**) CPT sensitivity of TopI in the sorted CD44+ and CD44− cells, respectively. S(hTopI) was incubated with whole cell extract from 10^4^ cells in presence of 15 µM, 30 µM, 60 µM CPT or DMSO as indicated in the figure. One example out of 12 individual microscopic images of each reaction sample is shown in the top panel, where red signals correspond to circularized S(hTopI) while green signals were generated from control circles. The lower panel shows a quantitative depiction of three independent experiments. For all REEAD experiments, to avoid misinterpretation due to potential uneven distribution of RCA primers printed on the surface, the number of red and green signals were counted on 12 microscopic images and the results depicted as the ratio between the number of red (R) TopI specific- and green (G) control circle generated signals (R/G) as described [45].

It has previously been reported that CSC like cells can be isolated from cell lines and that these cells are characterized by a rather drug insensitive phenotype [43]. We therefore set out to investigate how the CSC like cell population of Caco2 differs from the non-CSC subpopulation of the cell line with respect to sensitivity towards the anti-tumor drug CPT. First, the cells were stained with PE-conjugated CD44 or CD133 antibodies and the relative numbers of CD44+ and CD133+ cells were determined by FACS analyses. As evident from figure 1A, approximately 32% of the cells were CD44+ while close to 100% were CD133+. The number of CD44+ cells was reduced markedly (to 9%) by treatment with sodium butyrate (NaBt), which is a well know inducer of differentiation in cancer cell lines including Caco2 [44], [50], [51]. The number of CD133+ cells remained largely unchanged by this treatment (data not shown). These results are in agreement with previous work by Dalerbo *et al.*, suggesting that CD44 is a sufficient marker of CSCs in colorectal cancer [52]. We therefore decided to separate the cells solely on the basis of the CD44 expression. After staining and FACS separation the purity of the obtained cell populations was analyzed by immunophenotyping with CD44. As demonstrated in figure 1B the FACS separation resulted in 94% pure CD44+ and 99% pure CD44− cell populations, respectively.

To compare the CPT sensitivity of the two Caco2 cell subpopulations the FACS separated CD44+ and CD44− cell subpopulations (figure 1B) were treated with CPT concentrations ranging from 0 to 0.4 µM before survival was estimated in a standard MTT assay. The results were normalized to 100% survival for the untreated cells exposed to DMSO (used as a solvent for CPT) alone and depicted in the bar chart shown in figure 1C. As evident from the figure, the CD44+ cells were substantially less sensitive towards CPT treatment than were CD44− cells.

The CPT sensitivity of cells often correlates directly with the intracellular TopI activity level and the susceptibility of TopI towards the drug [11], [14]. Therefore, the activity and drug susceptibility of TopI in extracts from FACS sorted CD44+ and CD44− cells were measured directly using the previously described Rolling circle Enhanced Enzyme Activity Detection (REEAD) assay [45]. In this assay (see figure 1D) TopI cleavage-ligation is measured in terms of circularization of a dumbbell shaped DNA substrate (S(hTopI)). Each generated circle is then visualized at the single molecule level by subsequent rolling circle amplification (RCA) followed by hybridization of specific fluorescent probes to the generated RCA products as described [45], [53]. REEAD is an isothermal procedure where each TopI cleavage-ligation reaction results in one DNA circle, which in turn gives rise to a single visible fluorescent spot in the microscope. Hence, the assay allows highly sensitive analyses of TopI activity using very little cell material [54]. Reproducible quantitative data are easily obtained by normalization of TopI specific signals to signals obtained from a known concentration of spike-in control circles simply by calculating the ratio between TopI specific signals and signals resulting from the spike-in control circles as described in [45].

Figure 1E shows the results obtained when measuring the TopI activity in extracts from decreasing numbers (corresponding to 10^4^, 10^3^, 10^2^ or 10 cells as indicated in the figure) of the FACS sorted CD44+ or CD44− cells using the REEAD assay. The top panel shows representative examples of the microscopic views obtained in this experiment. The red signals represent the RCA products generated from circularized S(hTopI) resulting from TopI activity while the green signals represent RCA products generated from the spike-in control circles. The lower panel is a graphical depiction of the results obtained in three independent experiments where TopI activity is depicted as the ratio between TopI generated signals (red) and control circle generated signals (green). As evident from the chart, extract from CD44+ cells contained approximately three fold less TopI activity than did CD44− cells. This is in agreement with the decreased CPT sensitivity of CD44+ relative to CD44− cells observed in the survival experiment (figure 1C).

The CPT susceptibility of TopI expressed in the FACS sorted CD44+ or CD44− cells was compared by assaying TopI activity present in extracts from 10^4^ cells of either cell population in the presence of increasing concentrations of CPT. As evident from the graphical depiction of the results shown in figure 1F, TopI in extract from CD44+ cells was hardly affected by the utilized concentrations of CPT, whereas TopI in extract from CD44− cells was almost fully inhibited by 60 µM of CPT.

Based on these experiments we concluded that the decreased CPT sensitivity of CD44+ relative to CD44− Caco2 cells correlated with a relatively low enzyme activity and a CPT resistant phenotype of TopI expressed in these cells.

To address the possibility that the above effects could be an artifact arising as a result of the cell sorting procedure, we took advantage of the fact that cultivation of Caco2 cells in the presence of NaBt causes a major conversion of CD44+ cells to CD44− cells (data not shown and [44], [50], [51]). NaBt treatment caused a three-fold steady-state reduction in the proportion of CD44+ cells in our cultures. This was accompanied by proportionate increases in (i) cellular CPT sensitivity, (ii) TopI activity in cell extract, and (iii) CPT sensitivity of extracted TopI (data not shown). Thus, our findings with sorted cells are qualitatively recapitulated with unsorted cells.

### Activity and CPT sensitivity of TopI purified from CD44+ or CD44− Caco2 cells

The activity and CPT susceptibility of TopI can be envisioned modified by post-translational modifications, interactions to other proteins or mutations in the gene encoding the enzyme [55]. It is highly unlikely if not impossible that mutations account for different CPT susceptibility of TopI in dynamic subpopulations i.e. CD44+ and CD44− cells of the same cell line (in this case Caco2).

**Figure 2:**
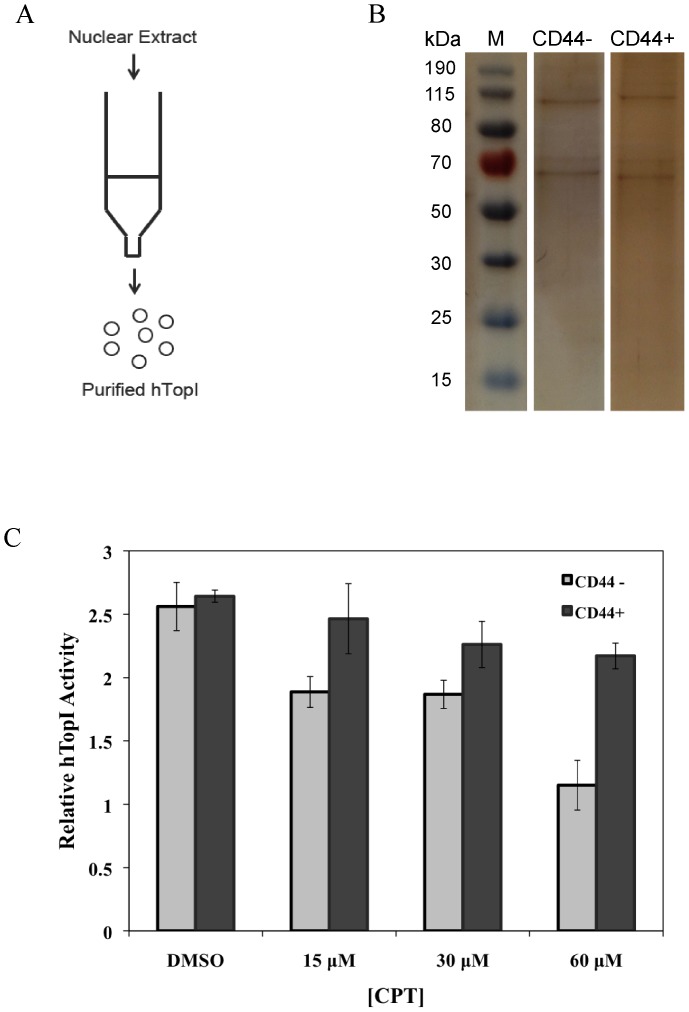
CPT sensitivity of TopI purified from nuclear extract of FACS sorted CD44+ or CD44− cell subpopulations. (**A**) Graphical illustration of column purification. (**B**) Shows the results of SDS phage followed by silver stain visualization of the purified enzyme fractions. Lane 1, is a size marker, lanes 2 and 3 show the enzyme fractions purified from CD44− and CD44+ cells, respectively. (**C**) Shows the CPT sensitivity of the purified enzyme as measured by REEAD. S(hTopI) was incubated with TopI purified from the CD44+ or CD44− cells in the presence of DMSO or 15 µM, 30 µM or 60 µM CPT as indicated. The results of three independent experiments were quantified and depicted as a bar chart as described in the legend of figure 1.

Therefore to elucidate if post-translational modifications or protein-protein interactions are the most likely mechanism behind the altered CPT susceptibility of TopI in CD44+ versus CD44− cells the endogenously expressed TopI in each cell subpopulation was separated from other proteins present in the cell extracts by Ni^2+^-column chromatography. Due to a histidine rich region in the N-terminal domain of TopI the wild-type enzyme exhibit high affinity for the Ni^2+^ resin [56], [57]. This procedure (graphically illustrated in figure 2A) resulted in approximately 50% pure TopI fractions, with very similar pattern of contaminating proteins for enzyme fractions originating from each cell subpopulation (see figure 2B). The purified enzyme fractions were analyzed with regard to CPT sensitivity using the REEAD assay. Figure 2C is a graphical depiction showing the result of three independent experiments where the activity of 5 ng of TopI purified from either CD44+ or CD44− cells were assayed in the presence of CPT concentrations ranging from 0 to 60 µM. As evident from the figure, the activity of TopI purified from each cell subpopulation was comparable in the absence of CPT. In contrast, the CPT insensitive phenotype of TopI from CD44+ cells observed in crude cell extracts was maintained in the purified enzyme fractions (compare the black bars in figure 2C). Since the pattern of contaminant proteins appeared very similar in the two enzyme fractions this result strongly suggest post-translational modifications as one potential mechanism of regulating the CPT susceptibility of TopI in the cell subpopulations of Caco2. Note, however, that a potential effect of contaminant protein factors present in the purified enzyme fractions cannot be completely ruled out from these studies. The difference in CPT susceptibility of TopI from the two cell subpopulations was more pronounced in cell extracts (figure 1F) suggesting that indirect factors such as protein-protein interactions also affects the CPT interaction.

### The phosphorylation patterns of TopI in CD44+ and CD44− cells differ

One of the well known post-translational modifications that can regulate the activity of TopI is phosphorylation [18], [58], [59], [60], [61]. We therefore investigated the phosphorylation pattern of TopI in extracts from different Caco2 cell subpopulations by 2D gel-electrophoresis.

**Figure 3:**
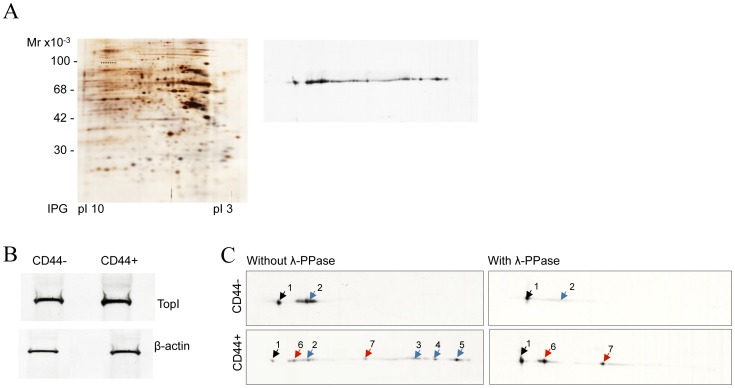
Phoshorylation pattern of TopI in extracts from CD44+ or CD44− cell subpopulations. (**A**) Two-dimensional silver staining (left panel) and immunoblot analysis (right panel) of TopI expression pattern in the Caco2 cell line. The positions of TopI are underlined. The TopI identity of two major isoforms recognized by TopI antibodies was ensured by the MS/MS analysis of several bands excised from the silver stained gel (data not shown). (**B**) One-dimentional immunoblot analysis of TopI expression in CD44− and CD44+ FACS sorted cell fractions by immunoblotting with TopI antibodies. β-actin was used as a loading control. (**C**) Two-dimentional immunoblot analysis of TopI expression and post-translational modification pattern in CD44− and CD44+ cell fractions (left-hand panels) by immunoblotting with TopI antibodies. Equal protein amounts were loaded on the gels. The time of blot exposure is identical. The resulting pattern of TopI after cell treatment with λ-PPase prior the 2D PAGE analysis is presented (right-hand panels). Arrowheads indicate multiple forms of TopI (black arrowheads – the position of the non-phosphorylated TopI, blue arrowheads – the positions of phosphorylated TopI isoforms; red arrowheads – the positions of non-phosphorylated otherwise modified isoforms). The positions of parental and modified TopI isoforms are verified by the superimposing of all analyzed blots.

Figure 3A shows the results of 2D gel-electrophoresis of extract from unfractionated Caco2 cells following visualization of proteins by silver staining (left panel) or Western blotting developed using an TopI specific antibody (right panel). As seen, the Western blot revealed a complex modification pattern of TopI in Caco2 cells with multiple isoforms, which are characterized by the same molecular mass (Mw) but highly different isoelectric points (pIs). All these spots were identified as TopI by MS analyses (data not shown).

To examine the phosphorylation profiles of the Top1 in the FACS separated CD44− or CD44+ cells, we exploited the fact that the removal of phospho-group(s) by treatment with λ-PPase alters the protein pI towards a more basic value as compared to the corresponding phosphorylated forms. The total protein amount of TopI was almost the same in extract from the two cell subpopulations, as confirmed by SDS PAGE analyses of extract from 0.5×10^5^ FACS sorted CD44+ or CD44− cells followed by Western blotting for visualization of TopI or actin (as a loading control) (see figure 3B).

The results of the dephosphorylation experiments are shown in figure 3C. As evident from the 2D gel-electrophoretic analyses CD44− cells expressed mainly two TopI isoforms with the same molecular weight (upper-left panel). Of these, the isoform in spot 1 with a more basic pI, presumably represented the non-modified TopI (black arrow) while the isoform in spot 2 with a more acidic pI most likely represented the phosphorylated variant of TopI (blue arrow). This interpretation was supported by the fact that the more acidic isoform almost entirely disappeared after treatment of cells with λ-PPase (compare upper-left and –right panels).

The pattern of the TopI isoforms in the CD44+ cells was more complex with a clear shift of at least 6 isoforms exhibiting differential intensities towards the acidic side (figure 3C, bottom-left panel spots 2–7, red and blue arrows). As seen in figure 3C (bottom–right panel), λ-PPase treatment resulted in the disappearance of 4 acidic spots (spots 2–5) suggesting that they corresponded to phosphorylated TopI isoforms.

The juxtaposition of protein blots from the CD44− and CD44+ cells revealed an exact matching of the position of unmodified TopI (spots 1) and one phosphorylated isoform (spots 2) in the two cell subpopulations. Note that the phosphorylation level of the TopI isoform corresponding to the spot 2 was significantly higher in CD44− than in CD44+ cells (compare intensities of spots 2 in figure 3C, upper-left and bottom-left panels). Note also that two acidic TopI variants in CD44+ (spots 6 and 7, red arrowheads) represented modifications that are most likely not related to phosphorylation because λ-PPase treatment did not affect their positions. The characterization of these modifications is out of the scope of the present study and will be the subject of future investigations.

Taken together the results demonstrated the complexity of the TopI post-translational modification pattern and showed the presence of multiple phosphorylation isoforms with different modification degree in the two cell subpopulations of Caco2. Hence, compared to TopI in CD44− cells, TopI in CD44+ cells were characterized by a markedly different phosphorylation pattern.

### The phosphorylation state regulates activity and CPT susceptibility of TopI present in cell extract from CD44+ or CD44− cells from the Caco2 cell line

**Figure 4:**
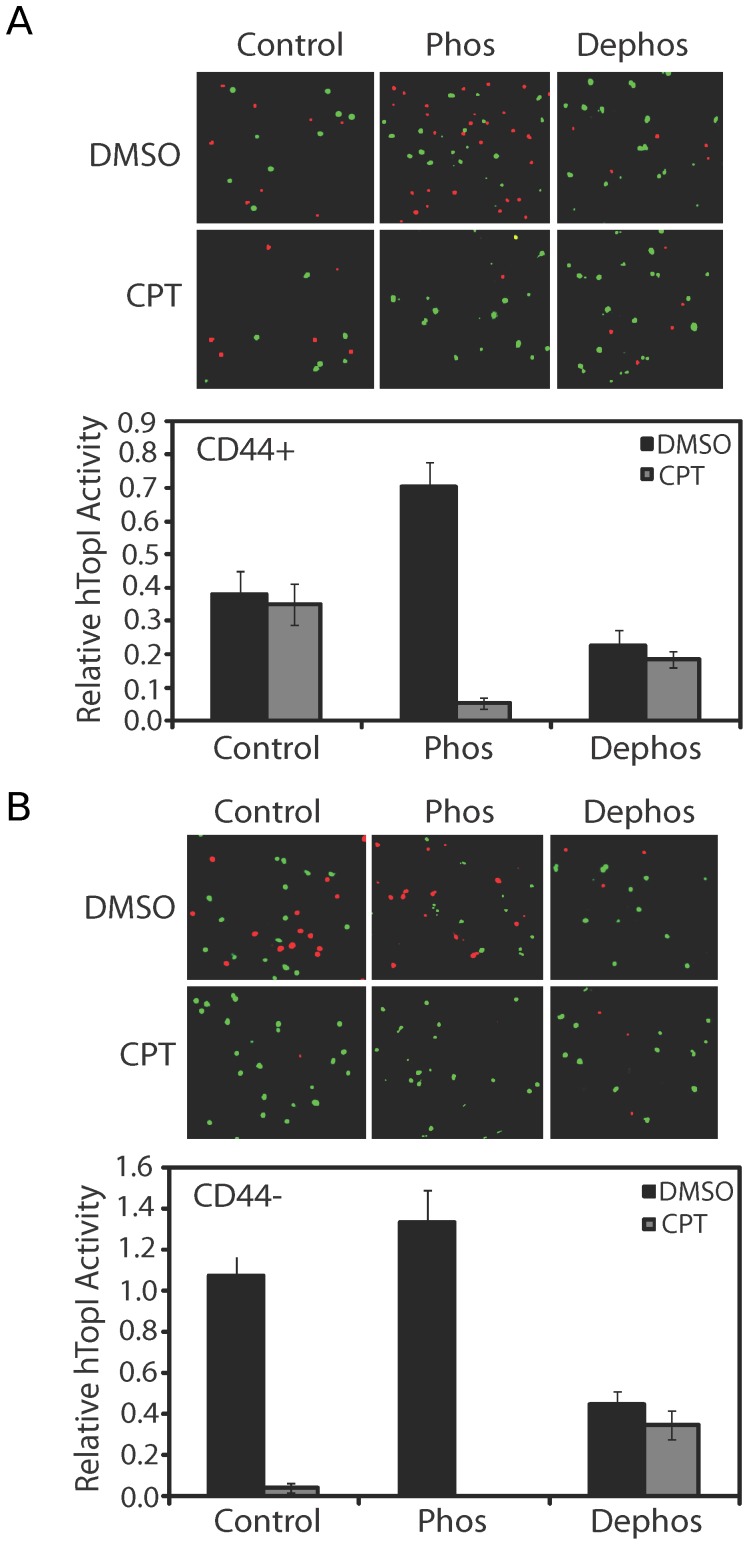
The effect of phosphorylation or dephosphorylation on the CPT sensitivity of TopI activity in extracts from FACS separated CD44+ cells or CD44− Caco2 cells. (**A**) The TopI activity in CD44+ cell extracts after phosphorylation or dephosphorylation. Whole cell extract was either left untreated (indicated by Control) or incubated with phosphatase (indicated by Dephos) or CK2 (indicated by Phos) before TopI activity was measured by REEAD in the presence of 60 µM CPT or 5% DMSO as indicated in the figure. One example randomly picked out of 12 individual microscopic images of each reaction sample is shown in the top panel. A quantitative depiction of three independent experiments, obtained as described in the legend of figure 1 is shown in the lower panel. (**B**) Same as (**A**), except that the analyses were performed on extracts from CD44− cells.

To investigate whether the different activity levels and CPT susceptibilities of TopI in extracts from CD44+ or CD44− cells could be ascribed to the different phosphorylation patterns the effects of *in vitro* dephosphorylation or phosphorylation of TopI in extract from the FACS separated CD44+ or CD44− cell subpopulation were addressed. For each experiment whole cell extracts were prepared from 10^4^ CD44+ or CD44− cells. Subsequently, the cell extracts were incubated with or without phosphatase or the serine/threonine protein kinase CK2 (casein kinase II). This kinase has previously been demonstrated to phosphorylate TopI *in vitro* [18], [62], [63], [64] and *in vivo* and was more recently associated with hyperphosphorylation and up-regulation of TopI activity in certain cancers [18], [63]. Following this treatment the TopI activities were measured using REEAD in the absence or presence of CPT. As evident from figure 4A, treatment of extract from CD44+ cells with CK2 markedly increased the TopI activity level (compare the black bars in the bar chart) and CPT sensitivity (compare black and gray bars) relative to the untreated extract. Dephosphorylation reduced the overall TopI activity level and had little effect on the CPT sensitivity of TopI in extract from the CD44+ cells. In contrast, treatment of extract from CD44− cells with CK2 resulted in a modest increase of the overall activity level (figure 4B, compare black bars of lower panel) and CPT susceptibility (compare black and gray bars) of the enzyme. Dephosphorylation of this cell extract, on the other hand, reduced both the activity and CPT sensitivity of TopI markedly.

These results are in agreement with the decreased CPT sensitivity of CD44+ relative to CD44− cells being caused at least in part by a different phosphorylation pattern of TopI in the two subpopulations.

## Discussion

In the current study we have addressed the importance of TopI activity regulation as a molecular mechanism behind resistance of CSCs towards the CPT family of anticancer drugs using the human colorectal adenocarcinoma cell line Caco2 as a model. The rationale for choosing this cell line was i) colorectal cancer is among the cancer types treated with drugs of the CPT family e.g. irinotecan (CPT-11) and topotecan (trade name Hycamtin) [2], [65], ii) Caco2 has previously been demonstrated to contain a rather large fraction of CSCs even in growth media containing serum [44], and iii) the CSC surface markers of colorectal cancer including the Caco2 cell line are relatively well defined [44], [52], [66]. Hence, CD133 and CD44 have both been reported as markers of CSCs in colorectal cancer. Haraguchi *et al.* reported that the CD133+ CD44+ population may be the real cancer initiating cells of human colorectal cancer [44] while Dalerba *et al.* found that most CD44+ cells are also CD133+, suggesting that CD44+ is an adequate marker for CSC in colorectal cancer [52]. This was consistent with our results.

We demonstrate a CPT resistant phenotype of the CD44+ CSC subpopulation from the Caco2 cell line, which is not shared by the CD44− non-CSC subpopulation. We investigated whether the CPT resistance of CSCs could be ascribed to altered activities of the TopI enzyme expressed in CSCs relative to the enzyme expressed in non-CSCs. The overall TopI activity levels as well as the CPT susceptibility of TopI in extracts from Caco2 cell subpopulations were analyzed using the previously described REEAD assay [45], [53]. We found that extract from cell subpopulations enriched for CD44+ cells exhibited a markedly decreased TopI activity level compared to extract from CD44− enriched cell subpopulations. This difference was not ascribed to different expression levels since the protein amount of TopI in extracts from the two cell subpopulations were comparable as demonstrated by Western blotting. Even more noteworthy, TopI present in extract from CD44+ enriched cell subpopulations was considerably less sensitive towards CPT inhibition than was TopI present in cell extracts from CD44− cell enriched subpopulations. The decreased TopI activity level and CPT susceptibility in CD44+ enriched cell subpopulations correlated directly with the decreased CPT sensitivity of these populations relative to CD44− cells as demonstrated by cell survival assays.

There is considerable evidence that phosphorylation is critical to the regulation of TopI activity. Hence, TopI purifies as a phosphoprotein and several studies have demonstrated that its *in vitro* activity and/or CPT susceptibility can be modulated by phosphorylation or dephosphorylation [67], [68], [69], [70], [71]. Phosphorylation of serine residues have been demonstrated to correlate with *in vivo* activity and the cellular sensitivity to CPT [18], [63], [64], [67], [72], [73]. In particular protein kinase CK2 is suggested to play and important function in regulating cellular CPT response via *in vivo* phosphorylation of TopI [18], [63]. We therefore addressed if a different phosphorylation state of TopI in CD44+ versus CD44− cells could explain the observed differences with regard to TopI activity and CPT susceptibility. 2D gel electrophoretic analyses revealed a markedly different phosphorylation pattern of TopI in CD44+ versus CD44− cells. In addition unique post-translational modifications other than phosphorylation were observed for TopI from the CD44+ cells. However, the fact that the observed differences in activity and CPT susceptibility of TopI could be changed by *in vitro* phosphorylation or dephosphorylation of TopI in extracts from CD44+ or CD44− cells supported that it was the different phosphorylations states that account for these differences. More specifically, phosphorylation by CK2 increased the activity and restored CPT susceptibility of TopI in extracts from CD44+ cells, while dephosphorylation of TopI in extracts from CD44− cells decreased activity and rendered the enzyme insensitive toward CPT.

Taken together these data suggest that reduced phosphorylation at specific sites in TopI from CD44+ cells leads to a reduced CPT susceptibility as well as a decreased activity level of the enzyme. These characteristics are consistent with the CPT resistance of this cell population. The involved phosphorylation sites are most likely threonines or serines since both activity and CPT sensitivity of TopI in CD44+ cell extracts was increased by phosphorylation by the serine/threonine kinase CK2. The different activity levels of TopI from CD44+ and CD44− cells were only observed in cell extracts and not in purified enzyme fractions. This suggests that the phosphorylation dependent regulation of activity was mainly indirect via e.g. protein-protein interactions or complex formations. In contrast, the different CPT susceptibility of TopI was at least to some extent retained in purified protein fractions although the difference was more pronounced when analyzed in extracts. This indicates that phosphorylation may modulate CPT sensitivity of TopI both directly and indirectly.

Indirect mechanisms of phosphorylation dependent regulation of TopI has previously been reported by the Gjerset group showing that hyperphosphorylated TopI forms a complex with the p14ARF tumor suppressor and that p14ARF may play a role in modulating TopI activity and CPT sensitivity of cancer cell lines [60].

To our knowledge the present study is the first report of differences in TopI of the CSC and non-CSC subpopulations of a cancer cell line that can explain the CPT resistant phenotype of CSC. Moreover, it represents one of only a few examples of the phosphorylation state of TopI modifying not only activity but also the CPT susceptibility of the enzyme [59], [69]. CK2 have previously been associated with TopI hyperphosphorylation in cancer cell lines rendering these cells highly sensitive towards CPTs [18], [63]. The *in vitro* experiments reported in this study do support a role of CK2 in regulating TopI activity also in Caco2 cells. However, whether the phosphorylated phenotype of TopI in the CD44− cells of Caco2 is regulated by CK2 *in vivo* remain the subject of further investigation. Likewise, other mechanisms of drug resistance in CSCs such as elevated levels of ABC transporters or repair activities have been addressed by others [44] and are out of the scope of the current study.

## References

[1] Ferlay J, Steliarova-Foucher E, Lortet-Tieulent J, Rosso S, Coebergh JW, et al (2013) Cancer incidence and mortality patterns in Europe: estimates for 40 countries in 2012. European journal of cancer 49: 1374–1403. 23485231 10.1016/j.ejca.2012.12.027

[2] Grivicich I, Mans DR, Peters GJ, Schwartsmann G (2001) Irinotecan and oxaliplatin: an overview of the novel chemotherapeutic options for the treatment of advanced colorectal cancer. Brazilian journal of medical and biological research = Revista brasileira de pesquisas medicas e biologicas/Sociedade Brasileira de Biofisica [et al] 34: 1087–1103. 10.1590/s0100-879x2001000900001 11514832

[3] Wolpin BM, Mayer RJ (2008) Systemic treatment of colorectal cancer. Gastroenterology 134: 1296–1310. 18471507 10.1053/j.gastro.2008.02.098 PMC2528832

[4] Beretta GL, Perego P, Zunino F (2006) Mechanisms of cellular resistance to camptothecins. Current medicinal chemistry 13: 3291–3305. 17168852 10.2174/092986706778773121

[5] Goldberg RM, Sargent DJ, Morton RF, Fuchs CS, Ramanathan RK, et al (2004) A randomized controlled trial of fluorouracil plus leucovorin, irinotecan, and oxaliplatin combinations in patients with previously untreated metastatic colorectal cancer. Journal of clinical oncology : official journal of the American Society of Clinical Oncology 22: 23–30. 14665611 10.1200/JCO.2004.09.046

[6] Douillard JY, Cunningham D, Roth AD, Navarro M, James RD, et al (2000) Irinotecan combined with fluorouracil compared with fluorouracil alone as first-line treatment for metastatic colorectal cancer: a multicentre randomised trial. Lancet 355: 1041–1047. 10744089 10.1016/s0140-6736(00)02034-1

[7] Moreau LC, Rajan R, Thirlwell MP, Alcindor T (2013) Response to chemotherapy in metastatic colorectal cancer after exposure to oxaliplatin in the adjuvant setting. Anticancer research 33: 1765–1768. 23564831

[8] Eng WK, Faucette L, Johnson RK, Sternglanz R (1988) Evidence that DNA topoisomerase I is necessary for the cytotoxic effects of camptothecin. Molecular pharmacology 34: 755–760. 2849043

[9] Soret J, Gabut M, Dupon C, Kohlhagen G, Stevenin J, et al (2003) Altered serine/arginine-rich protein phosphorylation and exonic enhancer-dependent splicing in Mammalian cells lacking topoisomerase I. Cancer Res 63: 8203–8211. 14678976

[10] Beretta GL, Gatti L, Perego P, Zaffaroni N (2013) Camptothecin resistance in cancer: insights into the molecular mechanisms of a DNA-damaging drug. Current medicinal chemistry 20: 1541–1565. 23432590 10.2174/0929867311320120006

[11] Leppard JB, Champoux JJ (2005) Human DNA topoisomerase I: relaxation, roles, and damage control. Chromosoma 114: 75–85. 15830206 10.1007/s00412-005-0345-5

[12] Hertzberg RP, Busby RW, Caranfa MJ, Holden KG, Johnson RK, et al (1990) Irreversible trapping of the DNA-topoisomerase I covalent complex. Affinity labeling of the camptothecin binding site. The Journal of biological chemistry 265: 19287–19295. 2172250

[13] Christiansen K, Westergaard O (1996) The effect of camptothecin on topoisomerase I catalysis. Annals of the New York Academy of Sciences 803: 50–59. 8993500 10.1111/j.1749-6632.1996.tb26376.x

[14] Pommier Y (2006) Topoisomerase I inhibitors: camptothecins and beyond. Nat Rev Cancer 6: 789–802. 16990856 10.1038/nrc1977

[15] Strumberg D, Pilon AA, Smith M, Hickey R, Malkas L, et al (2000) Conversion of topoisomerase I cleavage complexes on the leading strand of ribosomal DNA into 5′-phosphorylated DNA double-strand breaks by replication runoff. Molecular and cellular biology 20: 3977–3987. 10805740 10.1128/mcb.20.11.3977-3987.2000 PMC85758

[16] Tsao YP, Russo A, Nyamuswa G, Silber R, Liu LF (1993) Interaction between replication forks and topoisomerase I-DNA cleavable complexes: studies in a cell-free SV40 DNA replication system. Cancer research 53: 5908–5914. 8261402

[17] Pommier Y (1993) DNA topoisomerase I and II in cancer chemotherapy: update and perspectives. Cancer chemotherapy and pharmacology 32: 103–108. 8387398 10.1007/BF00685611

[18] Bandyopadhyay K, Gjerset RA (2011) Protein kinase CK2 is a central regulator of topoisomerase I hyperphosphorylation and camptothecin sensitivity in cancer cell lines. Biochemistry 50: 704–714. 21182307 10.1021/bi101110e PMC3046806

[19] Burgess DJ, Doles J, Zender L, Xue W, Ma B, et al (2008) Topoisomerase levels determine chemotherapy response in vitro and in vivo. Proceedings of the National Academy of Sciences of the United States of America 105: 9053–9058. 18574145 10.1073/pnas.0803513105 PMC2435590

[20] Sugimoto Y, Tsukahara S, Oh-hara T, Isoe T, Tsuruo T (1990) Decreased expression of DNA topoisomerase I in camptothecin-resistant tumor cell lines as determined by a monoclonal antibody. Cancer research 50: 6925–6930. 2170010

[21] Jansen WJ, Zwart B, Hulscher ST, Giaccone G, Pinedo HM, et al (1997) CPT-11 in human colon-cancer cell lines and xenografts: characterization of cellular sensitivity determinants. International journal of cancer Journal international du cancer 70: 335–340. 9033637 10.1002/(sici)1097-0215(19970127)70:3<335::aid-ijc15>3.0.co;2-e

[22] Sorensen M, Sehested M, Jensen PB (1995) Characterisation of a human small-cell lung cancer cell line resistant to the DNA topoisomerase I-directed drug topotecan. British journal of cancer 72: 399–404. 7640225 10.1038/bjc.1995.345 PMC2034011

[23] Madelaine I, Prost S, Naudin A, Riou G, Lavelle F, et al (1993) Sequential modifications of topoisomerase I activity in a camptothecin-resistant cell line established by progressive adaptation. Biochemical pharmacology 45: 339–348. 8382060 10.1016/0006-2952(93)90069-9

[24] Li XG, Haluska P Jr, Hsiang YH, Bharti AK, Kufe DW, et al (1997) Involvement of amino acids 361 to 364 of human topoisomerase I in camptothecin resistance and enzyme catalysis. Biochemical pharmacology 53: 1019–1027. 9174116 10.1016/s0006-2952(96)00899-4

[25] Kjeldsen E, Bonven BJ, Andoh T, Ishii K, Okada K, et al (1988) Characterization of a camptothecin-resistant human DNA topoisomerase I. The Journal of biological chemistry 263: 3912–3916. 2831213

[26] Pommier Y, Pourquier P, Urasaki Y, Wu J, Laco GS (1999) Topoisomerase I inhibitors: selectivity and cellular resistance. Drug resistance updates : reviews and commentaries in antimicrobial and anticancer chemotherapy 2: 307–318. 11504505 10.1054/drup.1999.0102

[27] Moisan F, Longy M, Robert J, Le Morvan V (2006) Identification of gene polymorphisms of human DNA topoisomerase I in the National Cancer Institute panel of human tumour cell lines. British journal of cancer 95: 906–913. 16983402 10.1038/sj.bjc.6603361 PMC2360536

[28] Gongora C, Vezzio-Vie N, Tuduri S, Denis V, Causse A, et al (2011) New Topoisomerase I mutations are associated with resistance to camptothecin. Molecular cancer 10: 64. 21619602 10.1186/1476-4598-10-64 PMC3120799

[29] Tesauro C, Morozzo della Rocca B, Ottaviani A, Coletta A, Zuccaro L, et al (2013) Molecular mechanism of the camptothecin resistance of Glu710Gly topoisomerase IB mutant analyzed in vitro and in silico. Molecular cancer 12: 100. 24004603 10.1186/1476-4598-12-100 PMC3766703

[30] Alison MR, Lim SM, Nicholson LJ (2011) Cancer stem cells: problems for therapy? The Journal of pathology 223: 147–161. 21125672 10.1002/path.2793

[31] Emmink BL, Van Houdt WJ, Vries RG, Hoogwater FJ, Govaert KM, et al (2011) Differentiated human colorectal cancer cells protect tumor-initiating cells from irinotecan. Gastroenterology 141: 269–278. 21459094 10.1053/j.gastro.2011.03.052

[32] Sell S (2010) On the stem cell origin of cancer. The American journal of pathology 176: 2584–2494. 20431026 10.2353/ajpath.2010.091064 PMC2877820

[33] Lapidot T, Sirard C, Vormoor J, Murdoch B, Hoang T, et al (1994) A cell initiating human acute myeloid leukaemia after transplantation into SCID mice. Nature 367: 645–648. 7509044 10.1038/367645a0

[34] Singh SK, Hawkins C, Clarke ID, Squire JA, Bayani J, et al (2004) Identification of human brain tumour initiating cells. Nature 432: 396–401. 15549107 10.1038/nature03128

[35] Kim CF, Jackson EL, Woolfenden AE, Lawrence S, Babar I, et al (2005) Identification of bronchioalveolar stem cells in normal lung and lung cancer. Cell 121: 823–835. 15960971 10.1016/j.cell.2005.03.032

[36] Al-Hajj M, Wicha MS, Benito-Hernandez A, Morrison SJ, Clarke MF (2003) Prospective identification of tumorigenic breast cancer cells. Proceedings of the National Academy of Sciences of the United States of America 100: 3983–3988. 12629218 10.1073/pnas.0530291100 PMC153034

[37] Kondo T, Setoguchi T, Taga T (2004) Persistence of a small subpopulation of cancer stem-like cells in the C6 glioma cell line. Proceedings of the National Academy of Sciences of the United States of America 101: 781–786. 14711994 10.1073/pnas.0307618100 PMC321758

[38] Setoguchi T, Taga T, Kondo T (2004) Cancer stem cells persist in many cancer cell lines. Cell cycle 3: 414–415. 15004528 10.4161/cc.3.4.799

[39] Bao S, Wu Q, McLendon RE, Hao Y, Shi Q, et al (2006) Glioma stem cells promote radioresistance by preferential activation of the DNA damage response. Nature 444: 756–760. 17051156 10.1038/nature05236

[40] Liu G, Yuan X, Zeng Z, Tunici P, Ng H, et al (2006) Analysis of gene expression and chemoresistance of CD133+ cancer stem cells in glioblastoma. Molecular cancer 5: 67. 17140455 10.1186/1476-4598-5-67 PMC1697823

[41] Phillips TM, McBride WH, Pajonk F (2006) The response of CD24(−/low)/CD44+ breast cancer-initiating cells to radiation. Journal of the National Cancer Institute 98: 1777–1785. 17179479 10.1093/jnci/djj495

[42] Hirschmann-Jax C, Foster AE, Wulf GG, Nuchtern JG, Jax TW, et al (2004) A distinct “side population” of cells with high drug efflux capacity in human tumor cells. Proceedings of the National Academy of Sciences of the United States of America 101: 14228–14233. 15381773 10.1073/pnas.0400067101 PMC521140

[43] Abdullah LN, Chow EK (2013) Mechanisms of chemoresistance in cancer stem cells. Clinical and translational medicine 2: 3. 23369605 10.1186/2001-1326-2-3 PMC3565873

[44] Haraguchi N, Ohkuma M, Sakashita H, Matsuzaki S, Tanaka F, et al (2008) CD133+CD44+ population efficiently enriches colon cancer initiating cells. Ann Surg Oncol 15: 2927–2933. 18663533 10.1245/s10434-008-0074-0

[45] Stougaard M, Lohmann JS, Mancino A, Celik S, Andersen FF, et al (2009) Single-molecule detection of human topoisomerase I cleavage-ligation activity. ACS Nano 3: 223–233. 19206270 10.1021/nn800509b

[46] Hann CL, Carlberg AL, Bjornsti MA (1998) Intragenic suppressors of mutant DNA topoisomerase I-induced lethality diminish enzyme binding of DNA. The Journal of biological chemistry 273: 31519–31527. 9813066 10.1074/jbc.273.47.31519

[47] Gromov P, Celis JE, Gromova I, Rank F, Timmermans-Wielenga V, et al (2008) A single lysis solution for the analysis of tissue samples by different proteomic technologies. Molecular oncology 2: 368–379. 19383358 10.1016/j.molonc.2008.09.003 PMC5527781

[48] Celis JET S, Gromovo P (2006) Gel-Based Proteomics: High-Resolution Two-Dimensional Gel Electrophoresis of Proteins. Isoelectric Focusing (IEF) and Nonequilibrium pH Gradient Electrophoresis (NEPHGE). Cell Biology: A Laboratory Handbook: Elsevier. pp. 165–174.

[49] Celis JE, Moreira JMA, Gromov P (2006) Determination of Antibody Specificity by Western Blotting. Cell Biology: A Laboratory Handbook: Elsevier. pp. 527–532.

[50] Augeron C, Laboisse CL (1984) Emergence of permanently differentiated cell clones in a human colonic cancer cell line in culture after treatment with sodium butyrate. Cancer research 44: 3961–3969. 6744312

[51] Souleimani A, Asselin C (1993) Regulation of c-myc expression by sodium butyrate in the colon carcinoma cell line Caco-2. FEBS letters 326: 45–50. 8325387 10.1016/0014-5793(93)81758-r

[52] Dalerba P, Dylla SJ, Park IK, Liu R, Wang X, et al (2007) Phenotypic characterization of human colorectal cancer stem cells. Proceedings of the National Academy of Sciences of the United States of America 104: 10158–10163. 17548814 10.1073/pnas.0703478104 PMC1891215

[53] Andersen FF, Stougaard M, Jorgensen HL, Bendsen S, Juul S, et al (2009) Multiplexed detection of site specific recombinase and DNA topoisomerase activities at the single molecule level. ACS Nano 3: 4043–4054. 19950974 10.1021/nn9012912

[54] Juul S, Ho YP, Koch J, Andersen FF, Stougaard M, et al (2011) Detection of single enzymatic events in rare or single cells using microfluidics. ACS Nano 5: 8305–8310. 21936557 10.1021/nn203012q PMC3823540

[55] Proszek J, Roy A, Jakobsen AK, Frohlich R, Knudsen BR, et al (2013) Topoisomerase I as a biomarker: detection of activity at the single molecule level. Sensors 14: 1195–1207. 10.3390/s140101195 PMC3926610 24434877

[56] Knudsen BR, Straub T, Boege F (1996) Separation and functional analysis of eukaryotic DNA topoisomerases by chromatography and electrophoresis. Journal of chromatography B, Biomedical applications 684: 307–321. 8906479 10.1016/0378-4347(96)00152-1

[57] Rossi F, Labourier E, Forne T, Divita G, Derancourt J, et al (1996) Specific phosphorylation of SR proteins by mammalian DNA topoisomerase I. Nature 381: 80–82. 8609994 10.1038/381080a0

[58] St-Amant C, Lussier S, Lehoux J, Laberge RM, Boissonneault G (2006) Altered phosphorylation of topoisomerase I following overexpression in an ovarian cancer cell line. Biochemistry and cell biology = Biochimie et biologie cellulaire 84: 55–66. 16462890 10.1139/o05-157

[59] Staron K, Kowalska-Loth B, Szumiel I (1996) Lowered phosphorylation of topoisomerase I is a direct reason for reduced sensitivity of L5178Y-S cells to camptothecin. Annals of the New York Academy of Sciences 803: 321–323. 8993529 10.1111/j.1749-6632.1996.tb26406.x

[60] Bandyopadhyay K, Lee C, Haghighi A, Baneres JL, Parello J, et al (2007) Serine phosphorylation-dependent coregulation of topoisomerase I by the p14ARF tumor suppressor. Biochemistry 46: 14325–14334. 18004878 10.1021/bi7013618

[61] Yu D, Khan E, Khaleque MA, Lee J, Laco G, et al (2004) Phosphorylation of DNA topoisomerase I by the c-Abl tyrosine kinase confers camptothecin sensitivity. The Journal of biological chemistry 279: 51851–51861. 15448168 10.1074/jbc.M404396200

[62] Kowalska-Loth B, Girstun A, Derlacz R, Staron K (2003) Activation of human topoisomerase I by protein kinase CK2. Molecular biology reports 30: 107–111. 12841581 10.1023/a:1023942226954

[63] Bandyopadhyay K, Li P, Gjerset RA (2012) CK2-mediated hyperphosphorylation of topoisomerase I targets serine 506, enhances topoisomerase I-DNA binding, and increases cellular camptothecin sensitivity. PloS one 7: e50427. 23185622 10.1371/journal.pone.0050427 PMC3503890

[64] Turman MA, Douvas A (1993) A casein kinase type II (CKII)-like nuclear protein kinase associates with, phosphorylates, and activates topoisomerase I. Biochemical medicine and metabolic biology 50: 210–225. 8260198 10.1006/bmmb.1993.1063

[65] Gerrits CJ, de Jonge MJ, Schellens JH, Stoter G, Verweij J (1997) Topoisomerase I inhibitors: the relevance of prolonged exposure for present clinical development. British journal of cancer 76: 952–962. 9328159 10.1038/bjc.1997.491 PMC2228255

[66] Ricci-Vitiani L, Lombardi DG, Pilozzi E, Biffoni M, Todaro M, et al (2007) Identification and expansion of human colon-cancer-initiating cells. Nature 445: 111–115. 17122771 10.1038/nature05384

[67] Pommier Y, Kerrigan D, Hartman KD, Glazer RI (1990) Phosphorylation of mammalian DNA topoisomerase I and activation by protein kinase C. The Journal of biological chemistry 265: 9418–9422. 2160979

[68] Samuels DS, Shimizu Y, Shimizu N (1989) Protein kinase C phosphorylates DNA topoisomerase I. FEBS letters 259: 57–60. 2557245 10.1016/0014-5793(89)81493-0

[69] Staron K, Kowalska-Loth B, Zabek J, Czerwinski RM, Nieznanski K, et al (1995) Topoisomerase I is differently phosphorylated in two sublines of L5178Y mouse lymphoma cells. Biochimica et biophysica acta 1260: 35–42. 7999792 10.1016/0167-4781(94)00175-3

[70] Staron K, Kowalska-Loth B, Szumiel I (1994) The sensitivity to camptothecin of DNA topoisomerase I in L5178Y-S lymphoma cells. Carcinogenesis 15: 2953–2955. 8001262 10.1093/carcin/15.12.2953

[71] Kaiserman HB, Ingebritsen TS, Benbow RM (1988) Regulation of Xenopus laevis DNA topoisomerase I activity by phosphorylation in vitro. Biochemistry 27: 3216–3222. 2839226 10.1021/bi00409a014

[72] Cardellini E, Durban E (1993) Phosphorylation of human topoisomerase I by protein kinase C in vitro and in phorbol 12-myristate 13-acetate-activated HL-60 promyelocytic leukaemia cells. The Biochemical journal 291 Pt 1: 303–307. 8385936 10.1042/bj2910303 PMC1132517

[73] Coderoni S, Paparelli M, Gianfranceschi GL (1990) Phosphorylation sites for type N II protein kinase in DNA-topoisomerase I from calf thymus. The International journal of biochemistry 22: 737–746. 2169438 10.1016/0020-711x(90)90009-r

